# Occupational Therapy in the Treatment of Breast Cancer-Related Lymphedema: A Narrative Review

**DOI:** 10.3390/medsci14010139

**Published:** 2026-03-17

**Authors:** Ana Belén Jiménez-Jiménez, Irene Elvira-Pastor, Fernando Jesús Mayordomo-Riera, María Nieves Muñoz-Alcaraz

**Affiliations:** 1 Interlevel Clinical Management Unit of Physical Medicine and Rehabilitation, Reina Sofía University Hospital, Córdoba and Guadalquivir Health District, 14011 Córdoba, Spain; anab.jimenez.jimenez.sspa@juntadeandalucia.es (A.B.J.-J.); sr1marif@uco.es (F.J.M.-R.); 2 Maimónides Biomedical Research Institute of Cordoba (IMIBIC), Reina Sofia University Hospital, University of Córdoba, 14004 Córdoba, Spain; 3 Department of de Applied Physics, Radiology and Physical Medicine, University of Córdoba, 14004 Córdoba, Spain; 4 Faculty of Medicine and Nursing, University of Córdoba, 14004 Córdoba, Spain; 5 Faculty of Medicine and Nursing, University of Málaga, 29071 Málaga, Spain; iep271002@gmail.com

**Keywords:** breast cancer-related lymphedema, manual lymphatic drainage, ULL volume reduction and occupational therapy

## Abstract

**Background/Objectives:** Breast Cancer-Related Lymphedema (BCRL) is one of the most prevalent complications among patients, causing physical limitations and a negative impact on their quality of life. Given its chronic nature and influence on personal autonomy, it is essential to review the therapeutic approaches applied to date. The main objective of this study was to analyze and to compare the effectiveness of the different treatments currently used in the management of BCRL, especially those that incorporate the intervention of an occupational therapist. **Methods:** A narrative review of the scientific literature published between 2013 and 2025 was conducted. The search was carried out in the PubMed, Scopus, Web of Science, and Dialnet databases. Inclusion and exclusion criteria were applied to select studies with therapeutic interventions, selecting eight studies for review. **Results:** Complex Decompression Therapy (CDT) is currently the standard treatment, although one of its components, manual lymphatic drainage, is controversial in terms of its effectiveness. Interventions such as Activity-Oriented Proprioceptive Anti-Edema Therapy (TAPA), adapted physical exercise, and hydrotherapy showed significant benefits in quality of life, functionality, and reduction in the volume of lymphedema. **Conclusions:** The therapeutic approach to BCRL must be multidisciplinary and personalized. Occupational Therapy (OT) provides a person-centered approach that contributes to improving occupational performance and patient well-being. More studies with greater methodological rigor and sample size are needed to unify clinical criteria.

## 1. Introduction

The lymphatic system is a part of the immune system whose main function is to transport lymph, maintain body fluid balance, and provide adaptive immune response [1]. It is composed of lymphatic vessels, responsible for collecting excess lymph from tissues and returning it to venous circulation; lymph nodes, encapsulated organs located along the path of the lymphatic vessels in strategic regions of the body, responsible for filtering the lymph of lymphatic vessels; and lymph, a clear fluid derived from interstitial plasma, rich in proteins and immune cells [2]. When the return of lymph is interrupted, retention of lymphatic fluid occurs in tissues, leading to generalized inflammation of the affected area. This condition is known as “lymphedema”. There are two types of lymphedema: primary, an underdevelopment of the lymphatic system, and secondary, an acquired obstruction in the lymphatic vessels. Secondary lymphedema is more common than primary. This complication usually appears during oncological processes because lymph nodes are one of the first structures reached by tumor cells that spread through the lymph [3].

For this reason, the evaluation of lymph nodes could be important for assessing the extent of a cancerous process. Therapeutic or surgical interventions on the lymph nodes can result in an interruption or blockage of lymphatic drainage and, therefore, increase the risk of developing secondary lymphedema [2]. However, the current management of breast cancer has envolved toward axillary surgical de-escalation in selected patients. The ACOSOG Z0011 trial demonstrated that omitting complete axillary lymphadenectomy in patients with limited sentinel node involvement does not compromise long-term survival [3]. A recent update to the ASCO guidelines supports the individualized use of sentinel node biopsy and the avoidance of extensive axillary surgery when clinically indicated, reducing treatment-related morbidity, including the risk of Breast Cancer-Related Lymphedema [4].

In certain subgroups of elderly patients and early-stage tumors with low biological risk, current guidelines even consider omitting sentinel node biopsy when lymph node information does not change the indication for systemic or radiation therapy, thus reinforcing the approach of axillary surgical de-escalation [5,6].

The most recent evidence confirms that the development of Breast Cancer-Related Lymphedema is multifactorial. Among treatment-related factors, axillary lymphadenectomy and regional radiotherapy remain the most consistent determinants. In terms of patient-related factors, high body mass index is the most robustly documented risk factor, while infections such as cellulitis and certain comorbidities have also been associated with increased risk [7,8].

The Spanish Society of Medical Oncology (SEOM) determined in 2020 that approximately 99% of breast cancer cases affect women, and between 0.5% and 1% of cases affect men, so the main risk factor is being female. This cancer represents a significant burden on health systems and patients who suffer from it [9].

The Spanish Association against Cancer states that, after breast cancer is treated with surgery in the armpit to remove the lymph nodes or nodules, there is a risk of up to 10% of developing lymphedema. If, in addition to surgery, radiotherapy is given in the armpit, the risk increases to 20–25% [10,11].

The symptoms of Breast Cancer-Related Lymphedema (BCRL) initially manifest at the physical level, especially in the extremities. The National Cancer Institute (NIH) notes that general symptoms of lymphedema include the following: heaviness, fullness, or tightness in the area of surgery or radiation therapy; swelling, numbness or tingling in affected area; discoloration and hardening of the skin; and cellulitis. People with lymphedema are at greater risk for cellulitis because skin in swollen areas, where it stretches and becomes thinner, making it easier for bacteria to enter and proliferate in areas of the body with excess fluid [12].

The physical manifestations of lymphedema, such as fluid accumulation in the tissues, cause restrictions in arm and shoulder mobility and reduction in strength. In addition, numbness, pain, and tingling aggravate functional limitation. Therefore, it is important to highlight not only the high incidence of this condition but also the consequences it can have on female survivors. Up to 30% of women may develop lymphedema secondary to breast cancer, significantly affecting their quality of life. As a result, this group of women is at a higher risk of psychological distress, up to 73% compared to those who do not develop this condition [13].

According to the International Classification of Functioning, Disability, and Health (ICF), BCRL can be understood as a condition that alters bodily functions and structures, potentially negatively impacting a person’s overall functioning [14]. In line with the American Occupational Therapy Association (AOTA) Framework for Occupational Therapy Practice [15], this functional and participatory impact justifies intervention by Occupational Therapy (OT), whose approach is geared toward promoting functional adaptation, autonomy, and participation from a person-centered approach in occupation and in the different contexts of daily life.

The International Society of Lymphology (ISL) classified limbs affected by lymphedema in the following stages:

− Stage 0 (subclinical): lymphedema is still latent and no changes are observed in the morphology of arm. Sometimes, patient reports symptoms such as heaviness or tightness, or sensory changes.
− Stage 1 (mild): fluid accumulation appears in affected limb and becomes visible. It decreases when the limb is kept elevated and may be reversible. Pitting edema is present.
− Stage 2 (moderate): swelling is more evident. The edema no longer subsides easily when the limb is elevated; skin begins to harden and thicken due to fibrosis. As a result, the pitting begins to disappear.
− Stage 3 (severe): also called “lymphostatic elephantiasis”. The swelling becomes excessive and irreversible, limiting activities of daily living. The skin becomes even thicker and harder, and there may be color changes or lymph secretions [16].

Complex Decompression Therapy (CDT) is currently recognized as the standard treatment for improving the essential functions of the lymphatic system in the context of Upper Limb Lymphedema by a large number of authors [17,18,19,20]

The ISL determines that CDT consists of two phases of application: the first is the “active phase,” in which the goal is to reduce swelling and improve physical function, while the second is the “maintenance phase,” in which the goal is to prevent the symptoms of this condition from worsening [19]. This treatment has four clinical practice modalities which, depending on the characteristics of patients, stage of lymphedema, and other variables that can be combined with the aim of achieving maximum functionality, depend on whether the first or second phase is being addressed. These clinical approaches are as follows:

− Compression therapy: bandages, sleeves, or other types of compression garments.
− Manual lymphatic drainage (MLD): precise, proportionate, and rhythmic maneuvers that activate and improve lymphatic circulation and promote the elimination of waste substances [21].
− Skin care: moisturizing creams, sun protection, and the use of protective clothing.
− Exercise therapy: stretching, strengthening, and postural exercises.
− Postural treatment: elevation of the limb to reduce the volume of lymphedema.

The advantages of CDT include a reduction in volume, pain, and heaviness in the arm; improved lymphatic drainage; reduction in episodes of cellulitis; and an acceptable improvement of quality of life [15,16,17,20]. In many patients, this treatment could be a useful option in order to avoid surgical treatments due to the limited evidence of their success [17].

Recent international consensus emphasizes that preventing cellulitis in CLL does not depend solely on the technical application of CDT but also on structured education and sustained self-management over time. The document from the International Society of Lymphology (ISL, 2023) highlights skin care and adherence to compression as pillars for reducing recurrent infections. From an Occupational Therapy perspective, establishing self-care habits and routines is a key intervention mechanism, in line with the Occupational Therapy Practice Framework (OTPF-4) and the American Occupational Therapy Association’s guidelines for cancer survivors, which identify performance patterns and the integration of treatment into daily life as determinants of sustainable outcomes [15,22,23,24].

Occupational Therapy (OT) plays a fundamental role in the treatment of BCRL, as it contributes to the restoration of lost or impaired functionality. Improving mobility and minimizing the discomfort caused by BCRL has a direct impact on patients’ quality of life, allowing them to return to their daily activities with as few limitations as possible. According to the Human Occupation Model (HOM), the physical alterations caused by lymphedema affect key components of occupation, such as volition, habituation, and performance capabilities, impacting not only the execution of activities but also the person’s motivation to participate in them [20].

On the other hand, and in accordance with the 4th Framework of the American Occupational Therapy Association (AOTA), this limitation in the functional capacity of patients has a direct impact on occupational performance, compromising areas such as Activities of Daily Living (ADL), social participation, and fulfillment of work roles. This leads to a decrease in the person’s autonomy and quality of life [15].

The role of OT in the management of BCRL is based on strategies to restore and optimize the functional independence of patients. To this end, applied interventions have the following objectives:

− Range of mobility: active and passive exercises to maintain and improve joint range of motion, prevent contractures, and reduce muscle stiffness.
− Strength and endurance: muscle strengthening programs to recover the lost strength without worsening the symptoms of lymphedema.
− Edema control: teach self-care techniques such as the proper use of compression garments, manual lymphatic drainage, and elevation postures to promote lymphatic return and reduce the volume of the affected arm.
− Training in ADL: facilitate the recovery of basic and complex skills for performing daily tasks such as dressing, cooking, working, and participating in recreational activities, taking into account the physical limitations of lymphedema.
− Education and psychosocial management: emotional support, stress management, and education to promote psychological and social adaptation.
− Environmental adaptation: ergonomic adjustments and assistive devices to facilitate daily activities and prevent injuries. Patient compliance and the ability to wear assistive devices are important [25].

In addition, as mentioned above, it is essential that occupational therapists collaborate with other health professionals to offer a comprehensive approach, including rehabilitation doctor, oncologists, physical therapists and nurses specialized in lymphedema. This interdisciplinary approach ensures that patients receive comprehensive care that addresses both the physical and emotional aspects of recovery.

## 2. Materials and Methods

BCRL is one of the most prevalent and functionally limiting complications for women who survive the disease. This condition affects them not only physically but also in their participation in meaningful activities, often reducing their quality of life and independence. Occupational Therapy aims to provide person-centered interventions that allow them to improve their independence and their performance in activities of daily living. The main objective of this review is to analyze and compare the effectiveness, functionality, and impact on quality of life of different therapeutic approaches, from Occupational Therapy, in the rehabilitation of BCRL.

We established the following specific objectives: to review the scientific literature on the different therapeutic approaches of BCRL; to identify the advantages and limitations of each type of approach; to compare the effects of each intervention on BCRL volume, pain, mobility, functionality, and quality of life; and to discuss areas of BCRL treatment that require further research and future development.

A detailed review of the literature was conducted in different scientific databases (PubMed, Scopus, Web of Science and Dialnet). The search was conducted using DeCS/MeSH terms in Spanish and English and combined Boolean operators (AND/OR). The search strategy included the following terms combined with Boolean operators: “Lymphedema” AND “Breast cancer”, “Occupational Therapy” AND “Breast cancer”, “Occupational Therapy” AND ‘Lymphedema’ OR “Terapia ocupacional” AND “Linfedema”. The search included articles published in the last twelve years, from 2013 to the present (April 2025), in Spanish or English and freely accessible.

The inclusion criteria were as follows: studies published in the last 12 years (between 2013 and 2025); articles in English or Spanish and freely accessible; clinical trials, systematic reviews, meta-analyses, observational studies and doctoral theses; articles on treatments following the development of BCRL; and studies with pre- and post-treatment variables (volume, pain, mobility, functionality and quality of life).

On the other hand, the exclusion criteria were as follows: publications without access to the full text; articles on BCRL prevention; publications on pharmacological treatments; and studies that did not include variables to quantify the results of treatment.

The selected studies were analyzed qualitatively and comparatively, focusing primarily on their impact on functionality, quality of life, treatment adherence, and the occupational approach of the interventions.

As this is a narrative review, no formal assessment of risk of bias or structured grading of certainty was performed. The methodology followed recommendations for narrative reviews (SANRA) [22]. For descriptive purposes, studies were classified according to the traditional hierarchy of levels of evidence proposed by the Oxford Centre for Evidence-Based Medicine (OCEBM), based on study design, with the aim of contextualizing the methodological strength of the available evidence, without performing quantitative synthesis [23].

## 3. Results

**Figure 1:**
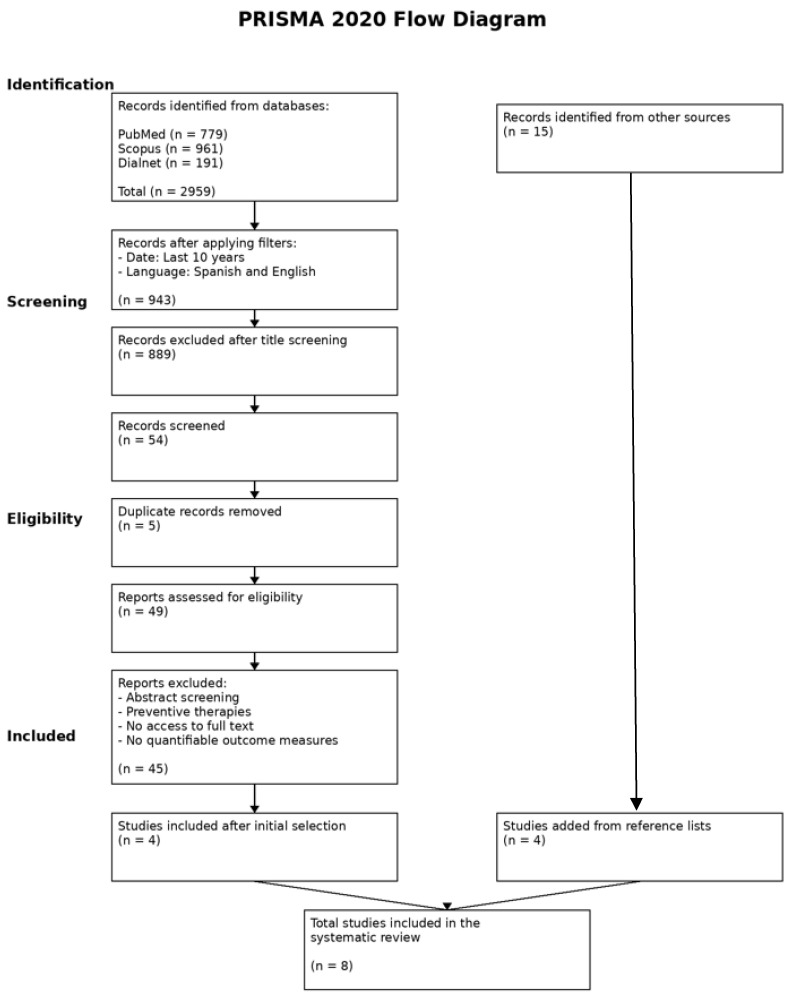
Flowchart.

A total of 2959 studies were identified after searching the databases. After removing duplicates and applying the inclusion and exclusion criteria, eight articles were selected for review. The included studies comprised clinical trials, systematic reviews, meta-analyses, observational studies, and doctoral theses. Figure 1 shows a flowchart of the search strategy developed by the author. Table 1 shows comparative characteristics of included studies on therapeutic approaches for BCRL, including clinical outcomes and axillary risk context.

## 4. Discussion

This narrative review synthesizes current evidence on therapeutic approaches for BCRL which, according to the International Society of Lymphology, affects more than one in five patients undergoing treatment for breast cancer [17].

In this context, the most recent consensus document from the International Society of Lymphology reaffirms that conservative treatment forms the basis of peripheral lymphedema management, maintaining Complex Decongestive Therapy (CDT) as the gold standard. This approach consists of an intensive phase, aimed at reducing volume, and a maintenance phase, in which compression garments, exercise, skin care, and self-care are used on a long-term basis.

This consensus highlights the need for a multidisciplinary approach involving specialized physicians, physical therapists, occupational therapists, and nursing staff trained in lymphology, emphasizing that therapeutic success depends not only on physical techniques but also on patient education, psychosocial support, and long-term adherence. The document also acknowledges emerging evidence supporting the safety and benefits of aerobic and resistance exercise in patients with breast cancer-associated lymphedema, as well as the potential of aquatic exercise as a complementary intervention, with no demonstrated increase in the risk of progression. These interventions are associated with functional improvements and improved quality of life.

The consensus also highlights the importance of incorporating functional and quality-of-life measures into outcome assessment, aligning with models focused on functioning and participation. However, the document also acknowledges the persistence of methodological limitations in the available literature and the need for studies with a higher level of evidence to enable more robust recommendations to be made [22].

Most of the included articles indicated that CDT is the default treatment for BCRL currently [17,18,19,20]. However, Muñoz-Alcaraz [26] highlighted several limitations about the CDT. Machado [19] et al. and Muñoz-Alcaraz [26] determined that there was a clear impact on adherence to CDT treatment by BCRL patients In this regard, Muñoz-Alcaraz [26] proposed to replace one of the traditional components of CDT, compression garments, with a proprioceptive cohesive bandage that does not adhere directly to the skin and minimizes tissue damage in patients with medical contraindications, intolerances, or aesthetic reasons. In the same way, the use of compression bandages could contribute to a decrease Upper Limb Lymphedema (ULL) and to an improvement Health-Related Quality of Life (HRQoL) in women after using the cotton bandages. Patients were instructed in the self-application of the bandage, thus promoting their autonomy in BCRL.

Additionally, Muñoz-Alcaraz [26] and Bills et al. [30] concluded that treatment with bandages and hydrotherapy resulted in an improvement in quality of life using the ULL-27 questionnaire administered at the beginning and end of both interventions. Significant improvements were reported in the social dimension in both cases and, in addition, the hydrotherapy treatment showed improvement in the emotional dimension 

At this point, Bills et al. [30] analyzed the effectiveness of hydrotherapy as a complementary therapy for the treatment of BCRL. Bills et al. took into account methodological limitations that the authors of the meta-analysis overlooked and refuted and overcame this approach, suggesting the need for further research into its effectiveness as a viable therapeutic alternative 

More recent studies with interventions such as Activity-Oriented Proprioceptive Anti-Edema Therapy (TAPA) and hydrotherapy have shown not only positive results in reducing volume and improved HRQoL but also greater therapeutic adherence [26,30]. These alternatives aim to overcome the limitations of traditional CDT and reinforce the need of personalized therapeutic approaches focused on adherence.

Another aspect that generated controversy in many of the studies included in this review was the effectiveness of one of the components of CDT, manual lymphatic drainage (MLD), on lymphedema volume. Domínguez et al. [31] considered that MLD did not offer additional benefits for reducing lymphedema volume. This supports the results obtained by O’Donnell et al. [28] who determined that MLD had a low level of recommendation despite being considered a safe and beneficial treatment as a complement to other therapies. This low recommendation was attributed to the limited validity of the research and the sample sizes.

Muñoz-Alcaraz [26] concluded that patients who received CDT and TAPA treatment had a significant clinical decrease in BCRL volume compared to baseline values, without significant statistically differences between groups. Similarly, Bergmann et al. [27] demonstrated that patients with MLD in their treatment and patients without MLD had a significant clinical decrease in volume, with no statistically significant differences. Therefore, it is important to design more specific studies focused on volume treatment because of the absence of consensus about the most effective intervention.

In contrast, Huang et al. [18] and Domínguez et al. [31] argued that it was unlikely that MLD produced a sufficient reduction in volume to be considered significant. However, the systematic review published by Ezzo et al. [17] confirmed MLD’s effectiveness when it was combined with another component of CDT, such as bandages.

This lack of consensus on the MLD treatment could be attributed, in part, to the limited quality and validity of many available studies. This may result in low-strength recommendations in clinical guidelines. O’Donnell et al. [28] highlighted the need to establish accurate clinical guidelines for the management of BCRL, noting that more research and consensus are required to define its use and applicability. The authors concluded that the current evidence is insufficient to recommend [26]. Baumann et al. [29] mentioned that the lack of definition of BCRL in medical care exacerbates this situation. The absence of clinical consensus not only complicates the interpretation of existing data but also hinders the possibility of further developing new studies 

From the Human Occupation Model, specific to Occupational Therapy, therapeutic adherence is understood as a process of habit consolidation (habituation) and volitional commitment (volition), influenced by environmental and contextual factors that condition the integration of self-management into daily life. Within this framework, the occupational therapist intervenes by facilitating the transformation of technical instructions—such as the use of compression or skin care—into stable and contextualized occupational routines, thus promoting the maintenance of results and the prevention of long-term complications [24].

The classification of studies according to level of evidence was performed for descriptive purposes, following the hierarchy of the Oxford Centre for Evidence-Based Medicine [32]. As this is a narrative review, no formal assessment of risk of bias or structured grading of certainty (e.g., GRADE) was performed [33]. In line with the 2023 Consensus of the International Society of Lymphology, our findings present methodological heterogeneity and should therefore be interpreted with caution [22].

This narrative review has several limitations that should be considered when interpreting its findings, such as the heterogeneity of the included studies in terms of design, sample size, interventions, and outcome measures, which makes it difficult to compare results and generalize conclusions. Some of the included studies used small samples and varied therapeutic protocols, contributing to a lack of consensus on the effectiveness of specific interventions, particularly with regard to manual lymphatic drainage. The inclusion of different types of study designs may affect the strength of the evidence.

In addition, many of the included studies do not stratify results according to patient-related risk factors, such as body mass index or infectious history, which may have influenced the magnitude of the observed effects and limited the comparability between different interventions.

These limitations point to the need for future research with greater methodological rigor, larger sample sizes, and more standardized outcome measures.

## 5. Conclusions

Lymphedema is a common and often chronic complication that negatively affects quality of life of breast cancer survivors. This narrative review has attempted to demonstrate the different therapeutic approaches used to deal with this condition and its management. In general terms, many included articles highlight that CDT is the current gold-standard treatment. However, there is no consensus among healthcare professionals about its use and benefits as treatment. On the other hand, the literature reviewed suggests that there are complementary interventions such as TAPA, physical exercise, and hydrotherapy that could provide significant benefits in volume reduction, improvement of functionality and quality of life. These interventions address dimensions not fully covered by CDT, such as therapeutic adherence or self-esteem.

One of the most remarkable findings has been the lack of consensus about the effectiveness of certain components of CDT, especially MLD. It has been concluded that, although no adverse or harmful effects have been reported in treatments, their validity remains questionable. Most authors mention the importance of reaching agreements in clinical practice and developing rigorous research with larger sample sizes.

In this context, OT is considered a key discipline in the treatment of BCRL. Its person-centered approach, focused on occupation and functional adaptation, allows professionals to offer treatments beyond physical interventions, considering psychosocial dimensions that are fundamental to the recovery and well-being of patients.

In conclusion, in order to provide effective, empathetic, and quality care in the treatment of BCRL, a multidisciplinary approach tailored to individual patient needs is essential. In line with the findings of the articles reviewed, it is essential to continue developing rigorous research that provides clear and up-to-date evidence on the most effective interventions in clinical practice. This process is a key step toward achieving consensus among healthcare professionals on treatments and interventions.

## Figures and Tables

**Table 1:** Comparative characteristics of included studies on therapeutic approaches for BCRL, including clinical outcomes and axillary risk context.

Study	Design (Level)	BCRL Stage	Intervention	Intensity/ Duration	Main Outcomes	Clinical Considerations	Risk Context/Axillary Management Considerations
**Muñoz-Alcaraz 2022** [26]	RCT (Level I)	Stage I–II	TAPA (proprioceptive cohesive bandage + activity-oriented functional training) vs. CDT	5 sessions/week; 4 weeks	Comparable volume reduction; greater HRQoL, shoulder mobility, and adherence in TAPA	Functional integration and self-management may enhance adherence and participation	Participants previously underwent axillary surgery; extent of ALND and radiotherapy likely influenced baseline risk. Obesity and adherence-related factors may modulate outcomes.
**Bergmann 2021** [27]	Narrative Review (Level V)	Not specified	CDT, exercise, electrotherapy, pneumatic compression, taping	Not standardized	CDT remains standard; exercise beneficial; inconsistent evidence for adjunct modalities	High heterogeneity limits protocol comparability	Identifies ALND and regional radiotherapy as major treatment-related risk factors; patient-related factors (BMI, infection) variably addressed.
**O’Donnell 2020** [28]	Systematic Review (Level I)	Not specified	CDT vs. MLD; guideline comparison	Variable across guidelines	CDT most frequently recommended; low consensus on MLD	Lack of standardized protocols and duration	Notes increasing surgical de-escalation (SLNB alone in selected cases), potentially reducing future BCRL incidence.
**Baumann 2018** [29]	Systematic Review (Level I)	Mixed stages	Physical exercise + standard care	2–5 sessions/week; 8–12 weeks (varied)	Volume reduction; improved symptoms and HRQoL	Exercise safe; protocol variability affects magnitude	No worsening observed even in patients post-ALND. Obesity may influence response and volume outcomes.
**Bills 2017** [30]	Systematic Review (Level I)	Not specified	Hydrotherapy + CDT vs. aquatic lymphatic therapy	2–3 sessions/week; 6–8 weeks (varied)	Volume reduction; improved pain and HRQoL	Water compression effect may enhance edema reduction; small samples	Surgical background heterogeneous; hydrostatic pressure beneficial regardless of axillary extent. Limited stratification by surgical risk.
**Domínguez 2017** [31]	Observational (Level III)	Mixed stages	Functional assessment (no intervention comparison)	Cross-sectional	Functional limitation associated with edema severity	Demonstrates relationship between volume and occupational performance	Surgical extent not stratified; highlights impact of severity rather than etiological risk factors.
**Ezzo 2015** [17]	Systematic Review (Level I)	Not specified	MLD + compression vs. compression alone	Variable	No significant additional volume reduction; some symptom improvement	MLD benefit unclear when isolated	Studies mainly included post-axillary surgery patients; heterogeneity in surgical staging limits subgroup analysis.
**Huang 2013** [18]	Systematic Review & Meta-analysis (Level I)	Mixed stages	CDT + MLD vs. CDT alone	Variable	No significant added volume reduction with MLD	Insufficient evidence for additive effect of MLD	Mixed surgical backgrounds; insufficient data to assess impact of extent of axillary intervention.

## Data Availability

No new data were created or analyzed in this study.

## Abbreviations

The following abbreviations are used in this manuscript:

ADL	Activities of Daily Living
AOTA	American Occupational Therapy Association
HRQoL	Health-Related Quality of Life
MLD	Manual Lymphatic Drainage
HOM	Human Occupation Model
ISL	International Society of Lymphology
BCRL	Breast Cancer-Related Lymphedema
NIH	National Cancer Institute
WHO	World Health Organization
TAPA	Activity-Oriented Proprioceptive Anti-Edema Therapy
CDT	Complex Decompression Therapy
OT	Occupational Therapy
ULL	Upper Limb Lymphedema
SEOM	Spanish Society of Medical Oncology
